# Remote regions, rapid recognition: performance and timeliness of BIOFIRE FilmArray BCID2 for diagnosing bloodstream infection in remote northern Australia

**DOI:** 10.1128/jcm.00311-26

**Published:** 2026-06-12

**Authors:** Bojana Simsic, Thomas Ewin, Jann Hennessy, Sonja Janson, Robert Baird, Ella M. Meumann

**Affiliations:** 1 Infectious Diseases Department, Division of Medicine, Royal Darwin Hospital, Northern Territory, Australia; 2 Microbiology Department, Territory Pathology, Darwin, Northern Territory, Australia; 3 Pharmacy Department, Royal Darwin Hospital, Darwin, Northern Territory, Australia; 4 Global and Tropical Health Division, Menzies School of Health Research, Charles Darwin University 10095, Northern Territory, Australia; Mayo Clinic Minnesota, Rochester, Minnesota, USA

**Keywords:** blood culture, bacteremia, bloodstream infection, rapid diagnostic test, nucleic acid amplification test, remote health

## Abstract

Bloodstream infection is a time-critical diagnosis; however, the vast geographic distances in rural and remote locations lead to prolonged specimen transport times, which can impact the timeliness and quality of results. In this retrospective observational study, we evaluated the performance and timeliness of the BIOFIRE FilmArray Blood Culture Identification 2 (BCID2) nucleic acid amplification test conducted on positive blood culture broth fluid in two very remote hospital laboratories in the Northern Territory, Australia, and compared the results to conventional testing done in central laboratories in the laboratory network. We reviewed 343 blood cultures, from which 366 organisms were identified, collected between January 2021 and August 2024. In 207/228 (90.8%) blood cultures with at least one pathogen, the clinically significant organism(s) were detected by BCID2. Concordance between BCID2 and conventional identification methods for organisms on the BCID2 panel was 98.3% (95% confidence interval 96.1%–99.4%). Seventy-four organisms isolated had no BCID2 target; of these, 21 were pathogens, 15 of which were *Burkholderia pseudomallei*. A total of 126 organisms were considered contaminants; 71 (56%) of these were detected by BCID2. Importantly, BCID2 detections preceded conventional identification results by a median of 2.8 days (interquartile range 2.0–3.1 days). The BCID2 panel performed well in the hands of general scientists and substantially improved the timeliness of bloodstream pathogen identification in our setting. The need for additional targets, including *B. pseudomallei* and common contaminants, was identified.

**IMPORTANCE:**

Bloodstream infection is a life-threatening condition requiring urgent treatment, and blood cultures are the mainstay of diagnosis. Timely incubation and processing of blood cultures maximize yield and clinical utility; however, prolonged specimen transport times in rural areas can impact the quality of results. We investigated the performance and timeliness of a rapid multiplex nucleic acid amplification test (the Blood Culture Identification 2 [BCID2] panel) for detection of bacteria and yeasts in positive blood culture broth fluid in two small remote Australian hospital laboratories. Formal organism identification and susceptibility testing were subsequently conducted in larger, central laboratories. We found that the BCID2 panel detected the clinically significant organism(s) in 90.8% of blood cultures with at least one pathogen, with 98.3% concordance between BCID2 and conventional identification methods for organisms on the panel. BCID2 detections preceded conventional identification by a median of 2.8 days. The assay substantially improved the timeliness of bloodstream pathogen identification in the two remote regions.

## INTRODUCTION

Sepsis is a time-critical medical emergency requiring urgent treatment to reduce the high mortality, which is more than 20% (1–4). Extremes of age, presence of co-morbidities, remote location, and low socio-economic status are all associated with increased incidence of sepsis (4). In Australia, those living in very remote areas have 1.7 times the rate of hospitalization for sepsis compared to those living in major cities (4). The Northern Territory (NT) of Australia covers an area of 1.42 million km^2^ with a population of approximately 255,000 people, representing only 1% of Australia’s total population. This makes it one of the least densely populated areas in the world. Thirty percent of the NT population identify as First Nations Australians, 74% of whom live in remote or very remote areas (5). A previous study found that sepsis incidence in the NT was five times higher than in temperate parts of Australia, with First Nations Australians disproportionately affected (6).

The current Australian Sepsis Clinical Care guidelines recommend starting appropriate antimicrobials within 60 min once sepsis is suspected, or if signs of infection-related organ dysfunction are present, and if possible after blood cultures have been collected (7). Empiric antibiotics target the most common pathogens for a given syndrome, are inevitably broad spectrum, and can have gaps in coverage; targeted therapy can be given once microbiology results are known. Targeted therapy should be the most appropriate agent for the given organism and syndrome, and reduces unnecessary exposure to broad-spectrum agents (8–10). The vast geographic distances and associated specimen transport times mean that in the NT, microbiology results can be delayed.

The BIOFIRE FilmArray Blood Culture Identification 2 (BCID2) panel detects 43 nucleic acid targets associated with bloodstream infection, including the Enterobacterales order, the *Staphylococcus* and *Streptococcus* genera, 23 bacterial species, 7 yeast species, and 10 bacterial antimicrobial resistance markers (11). It is performed on positive blood culture broth fluid, processing time is 1 h, and the assay has high diagnostic accuracy; it has been found to improve outcomes of bloodstream infection when combined with antimicrobial stewardship (11–15).

In this study, we aimed to evaluate BCID2 performance and time to results compared to phenotypic organism identification methods in two very remote hospital laboratories in the NT, Australia.

## MATERIALS AND METHODS

### Study setting and design

This retrospective observational study was conducted at Territory Pathology, the pathology provider for public hospitals in the NT, Australia. Royal Darwin Hospital (RDH) is located in the “Top End”; it is the largest NT hospital and is where the main Territory Pathology laboratory is located. There are six regional hospitals with branch laboratories belonging to the Territory Pathology network, including Alice Springs Hospital (ASH), the main referral center for Central Australia. The locations of the two smaller laboratories in our study were Gove District Hospital (GDH), which serves a population of ~14,600 in the 33,310 km^2^ East Arnhem region, and Tennant Creek Hospital (TCH), which serves a population of ~6,300 in the 322,710 km^2^ Barkly region (16). Both the GDH and TCH laboratories are staffed by multiskilled scientists who mainly perform hematological and biochemical testing and undertake limited microbiological testing. Scientific staff have a Medical Laboratory Science university qualification, and at least one staff member at each laboratory has ≥2 years of laboratory work experience. To minimize delays between blood culture collection and incubation, blood cultures are incubated on site at these two laboratories. When a bottle flags positive, wet preparation, Gram stain, and the BCID2 panel are performed on site and are communicated by telephone to a clinician. Further work-up is then done in a larger laboratory in the network (RDH or ASH).

The study included blood cultures with a BCID2 panel conducted and a microorganism detected or isolated from TCH from 15 January 2021 and from GDH from 26 January 2022 until 29 August 2024 at both sites. Blood culture episodes were excluded if (i) they were repeat blood cultures from the same hospital admission from which the same organism was isolated or (ii) they were within 5 days of collection of an initial positive blood culture from which the same organism was isolated.

Laboratory data for each blood culture were extracted from the laboratory information system (LIS), and clinical data for associated patients were extracted from electronic medical records. Data were stored in a Research Electronic Data Capture secure web-based database.

### Patient data

We collected demographic details for the patients from whom blood cultures had been collected, including date of birth, sex, Indigenous status, and location (GDH or TCH). Additional clinical data were collected for patients at the GDH site only, including onset of infection (community or hospital acquisition) and clinical syndrome. Community-acquired infection was defined as blood culture collected <48 h after admission, and hospital-acquired infection was defined as blood culture collected ≥48 h after admission. The clinical syndrome was defined as the most likely focus of infection, if known. We recorded the initial antibiotics administered, whether there was a change in antibiotics within 24 h of BCID2 result, and the nature of this, and the date and time of first appropriate antibiotics. Antimicrobial therapy was considered “appropriate” if it provided effective *in vitro* activity, even if the spectrum was unnecessarily broad. This classification aligns with the Australian Hospital National Antimicrobial Prescribing Survey definition of appropriateness, which encompasses both “optimal” and “adequate” prescribing. Under this framework, an antimicrobial prescription is deemed appropriate if it adheres to the Australian Therapeutic Guidelines (17) or local treatment guidelines, or is a reasonable alternative agent for the likely or confirmed causative pathogen(s) (18).

### Laboratory data

We collected laboratory data including the BCID2 result (organism(s) and resistance gene(s) detected), direct Gram stain result, phenotypic identification and antimicrobial susceptibility testing results, and whether the organism was considered a contaminant. Contaminants were defined as organisms deemed not pathogenic to the patient and were usually common skin or environmental flora with low virulence (19).

The date and time of entries for each stage of blood culture processing and communication of results were recorded, including blood culture collection, blood culture receipt, blood culture positivity in the incubator, Gram stain and BCID2 result communication to clinician, pathogen identification completion by phenotypic methods, antimicrobial susceptibility testing completion, and blood culture report finalization.

### Laboratory methods

Aerobic, anaerobic, and pediatric blood culture bottles collected at GDH and TCH were incubated on site using the BACT/ALERT automated blood culture system (bioMérieux). When the blood culture flagged positive, a wet preparation, Gram stain, and inoculation of chocolate agar were completed, and the BCID2 panel was conducted according to manufacturer’s instructions. Clinicians were notified by phone call of the results, and this communication was recorded in the LIS. The chocolate agar plate was placed in a sealed bag with a CO_2_ sachet and sent with the blood culture bottle to either RDH or ASH. Daily plane charters were used for transport from GDH to RDH (646 km flight taking 1 h 10 min), and transport from TCH was via bus to RDH (989 km taking 25 h) on Monday–Thursday and on Fridays and Sundays to ASH (509 km taking 9 h), with no specimen transport from TCH on Saturdays.

On arrival at RDH or ASH, further subculture of broth fluid on solid media and organism identification and susceptibility testing were conducted. VITEK 2 (bioMérieux) was used to undertake automated biochemical testing for organism identification (GP and GN cards) and antimicrobial susceptibility testing (P612 and N246 cards) at RDH and ASH. From May 2023, mass spectrometry using the VITEK MS (bioMérieux) became the primary organism identification method at RDH. Exceptions were identification of *Staphylococcus aureus*, which also incorporated tube coagulase, latex agglutination, and DNAse testing for identification, and beta-hemolytic streptococci for which Lancefield grouping was used (and with bacitracin susceptibility for confirmation of *Streptococcus pyogenes*), *Streptococcus pneumoniae* for which optochin susceptibility was used, and *Haemophilus influenzae* for which requirement for X and V factors was used. Coagulase-negative gram-positive cocci in clusters that were *S. aureus* latex agglutination-negative and considered contaminants were not routinely identified further, and if the colonial morphology was consistent, these were reported as “coagulase-negative *Staphylococcus*.” Coagulase-negative staphylococci were further identified using the VITEK MS on an *ad hoc* basis once this became available at RDH, but the result was not routinely made visible to clinicians. Where multiple skin commensals were isolated from a blood culture and were considered contaminants, these were reported as “mixed cutaneous flora.”

Susceptibility testing using Clinical & Laboratory Standards Institute (CLSI) breakpoints was conducted using the automated broth microdilution VITEK 2 system, with the addition of vancomycin gradient diffusion strip for *Staphylococcus aureus*, and Cepheid Xpert vanA/vanB and vancomycin gradient diffusion strip for *Enterococcus faecium*, and with the exception of *Streptococcus* spp., which were tested using CLSI disk diffusion methodology. Phenotypic testing for extended spectrum beta-lactamases (ESBLs) was not conducted. Enterobacterales with third-generation cephalosporin resistance were reported with a comment indicating “probable ESBL” if cefoxitin-susceptible and “probable plasmid-mediated AmpC with or without ESBL” if cefoxitin-resistant.

### Data analysis

Concordance between BCID2 and phenotypic identification was calculated as the number of concordant blood culture organism identification results divided by the total number of blood culture isolates, multiplied by 100 to convert to a percentage. Only isolates identified to the species level using phenotypic methods were included in the concordance calculation for *Staphylococcus epidermidis*. Key time points in the blood culture workflow were compared between the two study sites. These durations were not normally distributed, and the Mann-Whitney rank-sum test was used.

## RESULTS

A total of 2,130 blood cultures were collected at TCH and 1,592 blood cultures were collected at GDH during the study periods at the two sites. The BCID2 was conducted on 391 blood cultures. Of these, 16 had no organisms seen on Gram stain or isolated from culture and had a negative BCID2 result, and 32 were duplicate blood cultures with the same organism meeting exclusion criteria outlined in Materials and Methods. The remaining 343 blood cultures—227 from TCH and 116 from GDH—were included in the study.

The median age of the patients from whom blood cultures were collected was 51.5 years (interquartile range [IQR] 32–60 years), 49 patients (13.6%) were <15 years old, and 58 patients (16.7%) were >65 years old; 203 (58.7%) were female, and 307 (88.7%) were First Nations Australians. The clinical features of the patients from whom the 116 blood cultures at GDH were collected are presented in Table 1. Almost all (112/116, 96%) had community-onset infections, and the most common clinical syndromes were urinary tract infection (34/116, 26.1%), respiratory tract infection (28/116, 24.1%), and skin and/or soft tissue infection (15/116, 12.2%).

**TABLE 1:** Onset and clinical focus for patients at the GDH site

Clinical feature		Number (%)
Onset	Community	112 (96.6)
	Hospital	4 (3.4)
Clinical focus^*a*^	Urinary tract	34 (29.3)
	Respiratory tract	28 (24.1)
	Skin and/or soft tissue	15 (12.9)
	Central nervous system	9 (7.8)
	Other intra-abdominal site	8 (6.9)
	Bone and/or joint	4 (3.4)
	Liver and/or biliary tree	2 (1.7)
	Infective endocarditis	2 (1.7)
	Device-related	1 (0.9)
	No identifiable focus	13 (11.2)
	Other syndrome^*b*^	2 (1.7)
	Data missing	1 (0.9)

^*a*^ Three patients had more than one focus: one patient had a urinary tract infection and infection at another intra-abdominal site, one patient had respiratory and biliary tract infections, and one patient had a respiratory tract infection and acute rheumatic fever.

^*b*^ Two patients had suspected or confirmed acute rheumatic fever.

Of the total 3,722 blood cultures collected at the 2 sites, 126 (3.4%) had at least 1 probable contaminant isolated or detected by BCID2, and 228 (6.1%) had at least 1 pathogen isolated or detected by BCID2. In 207/228 (90.8%) blood cultures with at least one pathogen, the clinically significant organism(s) were detected by BCID2. Of 366 organism identifications in the 343 blood cultures, 287 (78.4%) were detected by BCID2 to the level of order (Enterobacterales), genus (*Staphylococcus* or *Streptococcus*), and/or species. The microorganisms isolated from the 343 blood cultures in the study are presented in Table 2 and Table 3 and are presented by study site in Tables S1 to S4. *Staphylococcus* spp. (*n* = 98) including *S. epidermidis* (*n* = 43) and *S. aureus* (*n* = 29), *Enterobacterales* (*n* = 96) including *Escherichia coli* (*n* = 80), and *Streptococcus* spp. (*n* = 78) including *Streptococcus pneumoniae* (*n* = 30) and *Streptococcus pyogenes* (*n* = 25) were the most common organisms identified by BCID2 and/or phenotypic methods. To quantify the burden of key pathogens in the two regions, the crude annual incidence was calculated and is presented in Table S5, noting that additional cases may have been diagnosed through other laboratories. Notably, in the Barkly region, the annual incidence of bloodstream infection with *S. pneumoniae* was estimated at 122.5 per 100,000 population and with *S. pyogenes* was 91.8 per 100,000 population.

**TABLE 2:** BCID2 and phenotypic identification results for organisms with a BCID2 target

	Organism	Number detected by BCID2	Number detected by BCID2 and identified phenotypically	Number identified phenotypically but not detected by BCID2	Concordance
Gram-positive	*Enterococcus faecalis*	2	2	0	2/2 (100%)
	*Enterococcus faecium*	1	1	0	1/1 (100%)
	*Staphylococcus* spp.	98	98	1^*a*^	98/99 (99%)
	*Staphylococcus aureus*	29	29	0	29/29 (100%)
	*mecA/C*- and MREJ-detected (MRSA)	15	15	0	15/15 (100%)
	*Staphylococcus epidermidis*	43	26^*b*^	0	26/26 (100%)
	*mecA/C*-detected (methicillin-resistant *S. epidermidis*)	33	–*^h^*	–	
	*Streptococcus* spp.	78^*c*^	76	0	76/78 (97%)
	*Streptococcus agalactiae*	1	1	0	1/1 (100%)
	*Streptococcus pneumoniae*	30	28^*d*^	0	28/30 (93%)
	*Streptococcus pyogenes*	25	25	0	25/25 (100%)
Gram-negative	*Acinetobacter baumannii* complex	3	3	0	3/3 (100%)
	Enterobacterales^*e*^	96	96	0	96/96 (100%)
	*Enterobacter cloacae*	3	3	0	3/3 (100%)
	*Escherichia coli*	79	79	1^*f*^	79/80 (99%)
	*E. coli* with CTX-M	14	–	–	
	*Klebsiella aerogenes*	1	1	0	1/1 (100%)
	*Klebsiella oxytoca* ^*g*^	1	0	0	0/1 (0%)
	*Klebsiella pneumoniae* group	9	9	0	9/9 (100%)
	*K. pneumoniae* with CTX-M	4	–	–	
	*Proteus* spp.	2	2	0	2/2 (100%)
	*Salmonella* spp.	2	2	0	2/2 (100%)
	*Haemophilus influenzae*	7	7	0	7/7 (100%)
	*Pseudomonas aeruginosa*	1	1	0	1/1 (100%)
Yeast	*Nakaseomyces glabratus (Candida glabrata)* complex	1	1	0	1/1 (100%)

^*a*^ Reported as coagulase-negative *Staphylococcus* but did not undergo species-level identification with automated biochemical testing or mass spectrometry.

^*b*^ Eighteen identified by BCID2 as *Staphylococcus epidermidis* were reported as coagulase-negative *Staphylococcus*, and one was reported as mixed cutaneous flora; these did not undergo species-level identification and were excluded from the concordance calculation.

^*c*^ Streptococci detected by BCID2 without a species-specific target were identified by phenotypic methods as *Streptococcus viridans* group (*n*=3), *Streptococcus anginosus* group (*n*=8), *Streptococcus gallolyticus* (*n*=2), Group G *Streptococcus* (*n*=4), Group C *Streptococcus* (*n*=1), and *Streptococcus *sp. not further identified (*n*=3).

^*d*^ Two *S. pneumoniae* isolates failed to grow.

^*e*^ One *Enterobacterales* isolate detected by BCID2 without a species-specific target was identified as *Pantoea* sp.

^*f*^ One *Escherichia coli* isolate was not detected by BCID2; however, the Enterobacterales and CTX-M targets were both positive.

^*g*^ *Klebsiella oxytoca *was detected by BCID2 in a polymicrobial blood culture with* K. pneumoniae, E. coli, and E. faecalis also isolated.*

^*h*^ –, not calculated.

**TABLE 3:** Organisms with no BCID2 target

	Organism	Number
Pathogens	*Aeromonas caviae*	1
	*Arcanobacterium haemolyticum^a^*	1
	*Burkholderia pseudomallei*	15
	*Enterococcus casseliflavus*	1
	*Enterococcus hirae*	1
	*Erysipelothrix rhusiopathiae*	1
	*Fusobacterium nucleatum*	1
Contaminants	*Alcaligenes* sp.	1
	*Bacillus* spp.	6
	*Candida colliculosa*	1
	*Corynebacterium* spp.	16
	*Cutibacterium* spp.	3
	*Gardnerella* sp.	1
	*Microbacterium* sp.	1
	*Micrococcus* spp.	14
	*Moraxella catarrhalis*	1
	*Penicillium* sp.	1
	*Sphingobacterium thalpophilum*	1
	*Veillonella* sp.	1
	Mixed cutaneous flora^*b*^	3
	Other gram-positive bacterium^*c*^	1
	Organism failed to grow^*d*^	1

^*a*^ Clinical presentation diabetic foot infection with *A. haemolyticum* +++ isolated from operative bone specimen.

^*b*^ Culture consisted of mixed skin flora, including organisms with morphology suggestive of* Micrococcus *sp. and *Corynebacterium *sp.

^*c*^ Anaerobic Gram-positive bacilli isolated, unable to be identified.

^*d*^ Gram-positive bacilli seen on Gram stain but no growth on subculture.

Concordance between BCID2 and phenotypic methods for identification of organisms on the BCID2 panel was 98.3% (95% confidence interval 96.1%–99.4%) (Table 2). Two *S*. *pneumoniae* isolates were detected by BCID2 and had typical Gram stain for this organism but failed to grow on subculture. One *E. coli* identified by the Vitek 2 GN card had the *Enterobacterales* and CTX-M but not *E. coli* target detected by BCID2; a further BCID2 test was done on the second bottle in the set when it became positive, and this was positive for the *Enterobacterales*, *E. coli*, and CTX-M targets. One *Klebsiella oxytoca* isolate was detected by BCID2 but not identified phenotypically from a polymicrobial blood culture with *Klebsiella pneumoniae*, *E. coli*, and *Enterococcus faecalis* also isolated and detected by BCID2; *K. oxytoca* was likely overgrown by the other organisms. Of 74 organisms isolated with no BCID2 target, only 21 (28.4%) were pathogens; 15 of these were *Burkholderia pseudomallei* and the remaining 6 were uncommon or rare organisms (Table 3). Overall, 126 organisms were considered contaminants; 71 (56.3%) of these were identified by BCID2. Common contaminants not detected by BCID2 included *Corynebacterium* spp. (*n* = 16), *Micrococcus* spp. (*n* = 14), *Bacillus* spp. (*n* = 6), and *Cutibacterium* spp. (*n* = 3).

Other than blood cultures reported as “mixed cutaneous flora,” 17 blood cultures were polymicrobial. Of these, 12 had 2 organisms isolated, 3 had 3 organisms isolated, and 1 had 4 organisms isolated. Thirty-five of 38 organisms in polymicrobial blood cultures were able to be identified by BCID2. Three organisms had no BCID2 target; these included an *Acinetobacter* sp., *Enterococcus casseliflavus*, and a *Corynebacterium* sp.

The time points of key events in the blood culture workflow are summarized in Table 4. For the 264 blood cultures with collection time documented, the median time from collection to receipt and placement in the incubator was 2.2 h (interquartile range [IQR] 0.5–11.6 h). Fifteen BCID2 results had no documentation of communication to a clinician, and/or no results recorded and were excluded from analysis of time to BCID2 results. The median time from blood culture flagging positive to BCID2 result was 1.4 h (IQR 1.1–2.0 h), from flagging positive to phenotypic identification was 68.3 h (IQR 48.1–73.9 h), and to reporting of antimicrobial susceptibility results was 71.2 h (IQR 49.6–90.3 h). Importantly, BCID2 detections preceded conventional identification results by a median of 2.9 days (IQR 2.0–3.5 days) at TCH and median 2.1 days (IQR 1.9–2.9 days) at GDH (*P* < 0.01).

**TABLE 4:** Duration between key events in the blood culture workflow^*a*^

Time from	Time to	Median (IQR) duration in hours, TCH (*n* = 227)	Median (IQR) duration in hours, GDH (*n* = 116)	*P* value	Median (IQR) duration in hours, both sites (*n* = 343)
Specimen collection	Incubation	2.6 (0.5–11.1)	1.7 (0.5–13.5)	0.8	2.2 (0.5–11.6)
Incubation	Flag positive	24.0 (20.7–27.5)	25.0 (20.7–44.3)	0.19	24.3 (20.7–35.5)
Flag positive	BCID2 result	1.3 (1.1–1.7)	1.8 (1.3-2.5)	<0.01	1.4 (1.1–2.0)
	Phenotypic identification	70.7 (48.8–77.1)	51.8 (47.0–71.2)	<0.01	68.3 (48.1–73.9)
	Antimicrobial susceptibility testing	72.1 (65.4–93.7)	64.9 (48.5–73.5)	<0.01	71.2 (49.6–90.3)

^*a*^ IQR, interquartile range; TCH, Tennant Creek Hospital; GDH, Gove District Hospital.

Of the 21 (of 96, 21.9%) *Enterobacterales* with third-generation cephalosporin resistance, 18 had the CTX-M target detected by BCID2 (Table 2 and Table S6). The median time from blood culture flagging positive to communication of the CTX-M result was 1.4 h (IQR 1.03–2.35 h), whereas third-generation cephalosporin resistance was reported at a median of 70.3 h (IQR 52.8–94.6 h) after the blood culture flagged positive. All 15 methicillin-resistant *S. aureus* (MRSA) isolates were detected by the BCID2 *mecA*/*C* and MREJ targets (Table 2 and Table S6), and for these 15, the median time from blood culture positivity to BCID2 result was 1.5 h (IQR 1.0–2.9), compared to a median of 68.8 h (IQR 57.6–71.9) from blood culture positivity to reporting of methicillin resistance. No other resistance mechanisms were detected by BCID2; however, a substantial proportion of Enterobacterales exhibited phenotypic resistance to antimicrobials used for empiric treatment of sepsis, including gentamicin (19/96, 19.8%) and ciprofloxacin (20/96, 20.8%) (Table S5). None were resistant to carbapenems.

Apart from MRSA and third-generation cephalosporin-resistant *Enterobacterales*, other organisms isolated were relatively susceptible. There were no vancomycin-resistant enterococci; all three *Acinetobacter baumannii* complex isolates were susceptible to gentamicin, ciprofloxacin, and meropenem; all 7 *Haemophilus influenzae* isolates were beta-lactamase-negative and susceptible to amoxicillin; and the 1 *Pseudomonas aeruginosa* isolate was susceptible to piperacillin/tazobactam, ciprofloxacin, ceftazidime, and gentamicin.

Antimicrobial treatment data were only available for the GDH site and were available for 100 patients. Forty-four of 100 patients (44%) had a change in antimicrobial therapy within 24 h of a BCID2 result: in 5/100 (5%), antimicrobial therapy was ceased; in 2/100 (2%), antimicrobials were initiated; in 19/100 (19%), a change was made to a narrower spectrum agent; in 16/100 (16%), therapy was changed to a broader spectrum agent; and in 2/100 (2%), a change was made, but the spectrum was similar to prior treatment. Five patients were changed from meropenem to a narrower spectrum agent for Enterobacterales without CTX-M detected, and vancomycin was ceased for 12 patients when BCID2 did not detect *mecA/C* or MREJ to indicate MRSA. Of six patients at GDH with CTX-M detected by BCID2, four had already received meropenem prior to the result being available, and for two, the antimicrobial treatment data were unavailable. All 10 patients at GDH with *mecA/C* and MREJ detected to indicate MRSA had received vancomycin prior to the BCID2 result being available.

The time of first appropriate antimicrobial therapy was available for 81 patients. Forty-nine patients (60.5%) received appropriate antimicrobials prior to BCID2 results. The time of first appropriate antimicrobial therapy was within 24 h after the BCID2 result in 17 (21.0%) patients; in all 17 instances, the organisms present were detected by the BCID2 panel. In the remaining 15 patients, appropriate antimicrobials were first administered >24 h after BCID2 result; in five instances, *B. pseudomallei* was subsequently identified; in three instances, the blood culture isolate was a contaminant; and other factors such as recall after discharge contributed to the remaining cases. It is important to note that the blood culture results were not always the main determinant for antimicrobial appropriateness; other clinical or microbiological data guided appropriate antimicrobial therapy in some cases.

## DISCUSSION

Diagnosis of bloodstream infection is time-critical, and timely incubation and processing of blood cultures are key to maximizing yield and clinical utility (20). The increasing use of high-throughput equipment and automation in microbiology laboratories is leading to increased centralization of services; however, in rural and remote areas, this has the potential to adversely impact the utility of blood culture results. Accordingly, we investigated the performance and timeliness of results of the BCID2 panel in two remote NT Australian hospital laboratories. Like many other rural laboratories, GDH and TCH have limited microbiology services and rely on centralized laboratories in Darwin and Alice Springs, 500–1,000 km away, for most microbiological testing. The implementation of BCID2 on site has allowed for substantially earlier identification of most bloodstream pathogens for patients in the associated remote hospitals.

The spectrum of pathogens observed in our study was similar to a previous NT study, with *S. aureus* and *E. coli* being the most common pathogens isolated (21). Although the populations in the regions served by the remote laboratories are small, we found that the incidence of several important pathogens, including *S. pneumoniae*, *S. pyogenes*, *S. aureus*, and *H. influenzae*, was substantially higher than the national average (21–24) and was particularly high for *S. pneumoniae* and *S. pyogenes* in the Barkly region served by TCH. Furthermore, 41.7% of *S. aureus* isolates were methicillin-resistant, and 21.9% of Enterobacterales were third-generation cephalosporin-resistant—representing the highest rates of each of these resistance patterns nationally (25). Each of these pathogens is associated with substantial morbidity and mortality, highlighting the importance of timely diagnosis in these two remote settings.

Overall, we found few discordances between BCID2 and culture of on-target pathogens, and the assay performed well in the hands of general scientists in the remote laboratories. Organisms detected by BCID2 but not through conventional methods included two *S*. *pneumoniae* isolates which failed to grow. Autolysis is well described for *S. pneumoniae*, particularly where there is a delay in processing (26, 27). This risk is mitigated by inoculation of chocolate agar on site in the remote laboratories, and it is possible that autolysis occurred in the bottles prior to processing. Additionally, one *K. oxytoca* isolate in a polymicrobial blood culture was likely overgrown by the two other Enterobacterales in the culture and was therefore unable to be identified. Polymicrobial blood cultures have previously been associated with BCID and BCID2 discordance with culture-based testing (28, 29), likely due to detection of organisms that are overgrown by other organisms in the bottle, or conversely failure to detect organisms present at a low level at the time the blood culture flags positive but which subsequently grow on subculture (30).

There were two instances where on-target organisms were not detected by BCID2: an *E. coli* isolate in an apparently monomicrobial culture in which the Enterobacterales and CTX-M targets only were detected and a coagulase-negative *Staphylococcus* also in a monomicrobial culture. The latter organism had been presumptively identified based on Gram stain and colonial morphology, as well as negative coagulase and latex agglutination tests, but did not get identified to the species level because it was not clinically significant. It is therefore possible that this was not a *Staphylococcus* species. There is not a clear explanation for the false-negative *E. coli* result; an organism with the same morphology from the second bottle in the set was positive for the *E. coli* target on BCID2, providing further support for an initial false-negative result.

The most important gap on the BCID2 panel in our setting is *B. pseudomallei*, which was isolated in 15 blood cultures at the GDH laboratory during the study period. Melioidosis has recently been found to be endemic in the southern United States, and the geographic boundaries of this pathogen—which already causes substantial morbidity and mortality across Asia and northern Australia—are expanding globally (31). *B. pseudomallei* is intrinsically resistant to many antimicrobials, and timely diagnosis is therefore critical for reducing the time to effective treatment. Early identification can also ensure the organism is handled using appropriate physical containment in the laboratory; in many jurisdictions, it is classified as a risk group three organism. *B. pseudomallei* would therefore be a valuable addition to the panel in many regions globally.

A second gap highlighted by our study is the lack of BCID2 targets for common contaminants. Timely detection of these may inform the decision to cease or commence antimicrobials, as well as the requirement to recall a patient to hospital if they have been discharged. Sometimes such patients have been discharged to remote communities, and recall requires aeromedical transfer, which is expensive and inconvenient if not needed, but likewise should not be delayed in the context of a bloodstream infection.

A previous study compared time to BCID results with conventional testing in a remote rural setting and found that time to results was reduced by 1.0 days (32). Similarly, we noted that the time from positivity to identification of on-target pathogens was significantly reduced from a median of 2.8 days using conventional methods to 1.35 h with BCID2. A substantial proportion of the time to finalize results through phenotypic testing in our setting comprises transport time due to the vast distances and very limited courier options, particularly for the Barkly region. Automated biochemical testing is slower than mass spectrometry and was the main organism identification method used for a substantial proportion of the study (and remains so in the Alice Springs Hospital laboratory), also contributing to delayed results.

Three previous pre-post intervention studies of BCID or BCID2 implementation showed reduced time to optimal antimicrobial therapy by 10–39 h (13, 15, 33), while other studies found no impact on the time to effective therapy but did find reduced duration of vancomycin for enterococcal bacteremia and more timely initiation of carbapenem therapy in patients with bloodstream infection due to ESBL-producing organisms (34, 35). A 2017 meta-analysis of 31 studies including 5,920 patients showed that molecular rapid diagnostic testing was associated with reduced time to effective therapy for bloodstream infection by a mean of 5 h (36), and a systematic review and network meta-analysis including 88 studies demonstrated significant reduction in mortality when rapid diagnostic tests (applied to positive blood culture broth or whole blood) were combined with antimicrobial stewardship (37). The retrospective nature and lack of a control arm meant that it was not possible to assess whether it was the BCID2 result or other factors that led to changes in antimicrobial therapy in our study, although changes to therapy were made in the 24 h following a result in a substantial proportion (44%) of patients with narrowing of the spectrum of cover in 18%, and appropriate therapy was first commenced in the 24 h following BCID2 result in 21%.

In conclusion, the BCID2 panel performed well in the hands of general scientists and substantially improved the timeliness of bloodstream pathogen identification in the two rural hospitals in our study. Addition of further targets on the BCID2 panel including *B. pseudomallei* and common contaminants would further increase the impact of this assay in our setting.

## SUPPLEMENTAL MATERIAL

Supplemental tables jcm.00311-26-s0001.pdf Tables S1 to S6.

ASM does not own the copyrights to Supplemental Material that may be linked to, or accessed through, an article. The authors have granted ASM a non-exclusive, world-wide license to publish the Supplemental Material files. Please contact the corresponding author directly for reuse.
