# Supplementary material for: Remote regions, rapid recognition: performance and timeliness of BIOFIRE FilmArray BCID2 for diagnosing bloodstream infection in remote northern Australia
Source: J Clin Microbiol. 2026 Jun 12;64(7):e00311-26. doi: 10.1128/jcm.00311-26 (PMC13343848; doi:10.1128/jcm.00311-26)
Supplement: Supplemental tables — Tables S1 to S6. [file jcm.00311-26-s0001.pdf]

Supplementary Appendix

Supplementary Table S1. GDH blood culture results: organisms with BCID2 target.

	Organism	Number detected by BCID2	Number detected by BCID2 and identified phenotypically	Number identified phenotypically but not detected by BCID2
**Gram positive**	*Staphylococcus* spp.	38	38	1[1]
	Staphylococcus aureus	17	17	0
	mecA/C and MREJ detected (MRSA)	10	10	0
	Staphylococcus epidermidis	13	8[2]	1
	mecA/C detected (MRSE)	9	-	-
	Streptococcus spp.	7	7	0
	Streptococcus pneumoniae	2	2	0
	Streptococcus pyogenes	4	4	0
**Gram negative**	*Acinetobacter baumannii complex*	3	3	0
	Enterobacterales	34	34	0
	Enterobacter cloacae	2	2	0
	Escherichia coli	27	27	0
	E. coli with CTX-M	5	-	-
	Klebsiella pneumoniae group	2	2	0
	K. pneumoniae with CTX-M	1	-	-
	Proteus spp.	1	1	0
	Salmonella sp.	2	2	0
	Haemophilus influenzae	1	1	0
	Pseudomonas aeruginosa	0	0	0
**Yeast**	*Nakaseomyces glabratus* (*Candida glabrata*) complex	1	1	0

[1] Reported as coagulase-negative *Staphylococcus*, not further identified

[2] Four isolates identified by BCID2 as *Staphylococcus epidermidis* were reported as coagulase-negative *Staphylococcus* and not identified further, and one was reported as mixed cutaneous flora

Supplementary Table S2. GDH blood culture results: organisms with no BCID2 target

	Organism	Number
**Pathogens**	*Arcanobacterium haemolyticum*[1]	1
	Burkholderia pseudomallei	15
	Enterococcus casseliflavus	1
	Enterococcus hirae	1
	Erysipelothrix rhusiopathiae	1
**Contaminants**	*Bacillus* spp.	2
	Candida colliculosa	1
	Corynebacterium spp.	5
	Cutibacterium sp.	1
	Gardnerella sp.	1
	Micrococcus spp.	5
	Mixed cutaneous flora[2]	3
	Other Gram-positive bacteria[3]	1

[1] Clinical presentation diabetic foot infection with *A. haemolyticum* +++ isolated from operative bone specimen

[2] Culture consisted of mixed skin flora including organisms with morphology suggestive of *Micrococcus* sp. and *Corynebacterium* sp.

[3] Anaerobic Gram-positive bacilli isolated, unable to be identified

Supplementary Table S3. TCH blood culture results: organisms with BCID2 target

	Organism	Number detected by BCID2	Number detected by BCID2 and identified phenotypically	Number identified phenotypically but not detected by BCID2
**Gram positive**	*Enterococcus faecalis*	2	2	0
	Enterococcus faecium	1	1	0
	Staphylococcus spp.	60	60	0
	Staphylococcus aureus	12	12	0
	mecA/C and MREJ detected (MRSA)	5	5	0
	Staphylococcus epidermidis	30	18[1]	0
	mecA/C detected (MRSE)	24	-	
	Streptococcus spp.	71	69	0
	Streptococcus pneumoniae	28	26[2]	0
	Streptococcus pyogenes	21	21	0
**Gram negative**	*Enterobacterales*	62	62	0
	Enterobacter cloacae	1	1	0
	Escherichia coli	52	52	1[3]
	E. coli with CTX-M	9	-	
	Klebsiella aerogenes	1	1	0
	Klebsiella oxytoca[4]	1	0	0
	Klebsiella pneumoniae group	7	7	0
	K. pneumoniae with CTX-M	3	3	
	Proteus spp.	1	1	0
	Haemophilus influenzae	6	6	0
	Pseudomonas aeruginosa	1	1	0

[1] Twelve isolates identified as *S. epidermidis* by BCID2 were reported as coagulase-negative *Staphylococcus* and not identified further

[2] Two failed to grow on subculture

[3] One *E. coli* isolate identified by the Vitek 2 Gram-negative identification card had the *Enterobacterales* and CTX-M but not *E. coli* target detected.

[4] *Klebsiella oxytoca* detected by BCID2 in a polymicrobial blood culture with *K. pneumoniae*, *E. coli*, and *E. faecalis* also isolated

Supplementary Table S4. TCH blood culture results: organisms with no BCID2 target

	Organism	Number
**Pathogens**	*Aeromonas caviae*	1
	Fusobacterium nucleatum	1
**Contaminants**	*Alcaligenes* sp.	1
	Bacillus spp.	4
	Corynebacterium spp.	11
	Cutibacterium spp.	2
	Microbacterium sp.	1
	Micrococcus spp.	9
	Moraxella catarrhalis	1
	Penicillium sp.	1
	Sphingobacterium thalpophilum	1
	Veillonella sp	1
	Organism failed to grow[1]	1

[1] Gram-positive bacilli seen on Gram stain but no growth on subculture

Supplementary Table S5. Estimated annual incidence of key pathogens.

**Bloodstream pathogen**	**Annual incidence per 100,000 population, Barkly region**	**Annual incidence per 100,000 population, East Arnhem region**
Staphylococcus aureus	52.5	51.3
Streptococcus pyogenes	91.8	12.1
Streptococcus pneumoniae	122.5	6.0
Burkholderia pseudomallei	0	45.3
Escherichia coli	227.4	81.5
Haemophilus influenzae	26.2	3.0

Supplementary Table S6. Phenotypic antimicrobial susceptibility results for Enterobacterales

	TCH (n, %)	GDH (n, %)	Total
**Total Enterobacterales**<br>*(E. coli, K. pneumoniae, K. oxytoca, K. aerogenes, Enterobacter cloacae, Pantoea sp.)*	62	34	96
Presence of CTX-M gene	12 (19.4%)	6 (17.6 %)	18 (18.8 %)
3rd generation cephalosporin resistance	13 (21.0%)	8 (23.5 %)	21 (21.9 %)
Gentamicin resistance	6 (9.7 %)	13 (38.2 %)	19 (19.8 %)
Ciprofloxacin resistance	6 (9.7 %)	14 (41.2 %)	20 (20.8 %)
